# Plant Phosphoglycerolipids: The Gatekeepers of Vascular Cell Differentiation

**DOI:** 10.3389/fpls.2016.00103

**Published:** 2016-02-12

**Authors:** Bojan Gujas, Antia Rodriguez-Villalon

**Affiliations:** Vascular Development Group, Department of Biology, Institute of Agricultural Science, Swiss Federal Institute of Technology in ZürichZürich, Switzerland

**Keywords:** phosphoinositides, vascular development, phloem, xylem, phosphatidylcholine, signal transduction, membrane

## Abstract

In higher plants, the plant vascular system has evolved as an inter-organ communication network essential to deliver a wide range of signaling factors among distantly separated organs. To become conductive elements, phloem and xylem cells undergo a drastic differentiation program that involves the degradation of the majority of their organelles. While the molecular mechanisms regulating such complex process remain poorly understood, it is nowadays clear that phosphoglycerolipids display a pivotal role in the regulation of vascular tissue formation. In animal cells, this class of lipids is known to mediate acute responses as signal transducers and also act as constitutive signals that help defining organelle identity. Their rapid turnover, asymmetrical distribution across subcellular compartments as well as their ability to rearrange cytoskeleton fibers make phosphoglycerolipids excellent candidates to regulate complex morphogenetic processes such as vascular differentiation. Therefore, in this review we aim to summarize, emphasize and connect our current understanding about the involvement of phosphoglycerolipids in phloem and xylem differentiation.

## Introduction

**FIGURE 1:**
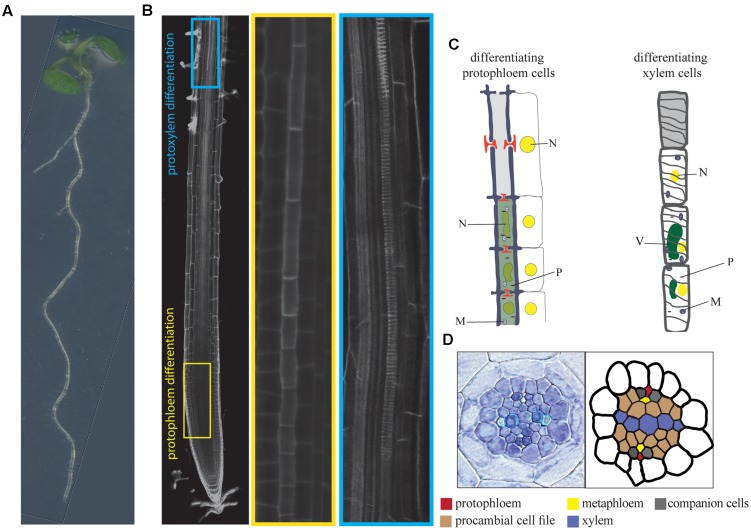
**Vascular differentiation in *Arabidopisis* roots.** **(A)** *Arabidopsis thaliana* seedling which root tip is shown in detail in **(B)**. **(B)** A propidium-iodide stained root tip imaged using confocal microscopy which differentiating protophloem (highlighted in yellow) and protoxylem (highlighted in blue) strands are magnified on the right panels. **(C)** Schematic representation of subcellular events associated to protophloem and xylem differentiation. **(D)** Radial organization of root stele. N: Nucleus; P: Plastids; M: Mitochondria; V: Vacuole.

As sessile organisms, plants have evolved signal transduction mechanisms to respond to a wide range of environmental and physiological signals to fine-tune their growth and development. In higher plants, such signals are mainly delivered by the vascular system, a long-distance transport network that connects distantly separated organs. In *Arabidopsis thaliana (Arabidopsis)*, the vascular system comprises two functionally specialized conducting elements: the xylem, required for the root-to-shoot transport of water and minerals, and the phloem, responsible for the reallocation of photosynthetic compounds from source-to-sink organs (**Figure 1A**). To become conductive elements, vascular cells undergo two drastic, albeit very different morphogenetic differentiation programs, which involve the degradation of nearly all their organelles. While protophloem cell clearance starts with nucleus degradation concomitant with cell wall thickening, the final maturation of xylem cells is initiated by the vacuolar rupture once the secondary cell wall is built (**Figures 1B,C**; Lucas et al., 2013; Schuetz et al., 2013; Furuta et al., 2014; Rodriguez-Villalon et al., 2014). At the end of the differentiation process, strands of lignified dead xylem cells will be formed, resulting in a continuous system of adjoining hollow cells transporting water and nutrients to the above-ground organs. On the contrary, enucleated protophloem cells will remain alive as metabolic needs will be provided by neighboring cells, the so-called companion cells (CC; **Figure 1D**). The latter will facilitate the loading of molecular cargo into the phloem stream, ensuring the long-distance transport of essential growth regulators (Oparka and Turgeon, 1999; Stadler et al., 2005). In addition to photoassimilates, phloem tissue delivers small RNAs, defense-related factors, peptides, and hormones throughout the whole plant body to regulate many aspects of plant development (Ruiz-Medrano et al., 2001). For example, recent studies have demonstrated that the phloem-mediated shoot-to-root transport of auxin is crucial in sculpting the architecture of root tissues (Rodriguez-Villalon et al., 2015). Moreover, phloem transport plays a pivotal role in the initiation of flowering events, as demonstrated by the translocation of *FLOWERING LOCUS T (FLT)* from CC to the phloem stream to reach the shoot apical meristem (Yoo et al., 2013). Surprisingly, beyond these well-characterized factors, recent studies have reported the presence of phosphoglycerolipids and lipid-transport proteins in vascular exudates, raising the question whether these compounds might act not only as membrane components but also as long-distance signaling factors (Guelette et al., 2012). Furthermore, phosphoinositides (PIs) are known to act as constitutive signals defining organelle identity and regulating subcellular trafficking (Janda et al., 2013). The ability of these lipids to be rapidly synthesized, modified and hydrolyzed implicates them as suitable candidates to signal complex cellular processes such as vascular differentiation. Therefore, in this review we will summarize our current understanding about the diverse roles of these metabolites as subcellular organizers during phloem and xylem development, with a particular focus on vascular cell differentiation.

## Phosphoglycerolipids Structure and Biosynthesis

**FIGURE 2:**
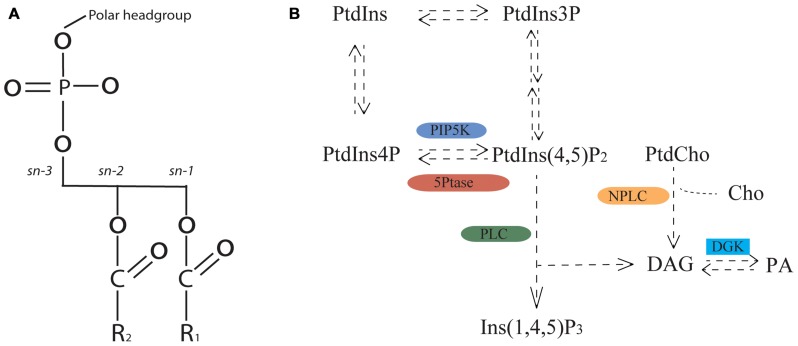
**Phosphoglycerolipid structure and biosynthetic pathway.** **(A)** Phosphoglycerolipid structure containing glycerol backbone, polar headgroup and fatty acids (represented by R1 and R2). Polar headgroup refers to choline, serine, ethanolamine or inositol. **(B)** Simplified view of the phosphoglycerolipid biosynthetic pathway. PtdIns, phosphatidylinositol; PtdIns3P, phosphatidylinositol 3-phosphate; PtdInsP(4,5)P_2_, phosphatidylinositol 4,5-bisphosphate; PtdIns4P, phosphatidylinositol 4-phosphate; PtdCho, phosphatidylcholine; Cho, choline; DAG, diacylglycerol; PA: phosphatidic acid; Ins(1,4,5)P_3_, inositol 1,4,5 triphosphate; PIP5K, PtdIns4P-5-KINASE; 5PTase, PHOSPHATIDYLINOSITOL 5′ PHOSPHATASE; PLC, PHOSPHOLIPASE C; NPC, NON-SPECIFIC PHOSPHOLIPASE C; DGK, DIACYLGLYCEROL KINASE.

The common structural feature of phosphoglycerolipids is a glycerol backbone that is acylated on hydroxyls at positions *sn*-1 and *sn*-2. The hydroxyl group at the third position can be esterified by a wide variety of alcohol-derived compounds ranging from *myo*-inositol or choline to ethanolamine or serine (**Figure 2A**). Interestingly, phosphoglycerolipids are structural compounds that exhibit dual functions in cell membranes: while their fatty acid tail is embedded in the membrane, their polar headgroup is exposed to the cytosol and display cell signaling functions (Di Paolo and De Camilli, 2006). Of particular interest are the signaling activities of PIs, which soluble *myo*-inositol-containing headgroup can be phosphorylated at different positions, giving rise to a broad spectrum of regulatory compounds (Munnik and Nielsen, 2011; Heilmann and Heilmann, 2015). In particular, the biologically active plant PIs are produced by the activity of PI-kinases, which reversibly phosphorylate at different positions the inositol headgroup. Importantly, each kinase reaction is mirrored by a PI-phosphatase catalyzing exactly the opposite enzymatic step (**Figure 2B**), ensuring the fast conversion among different PI species. The most abundant PI in plant cells is phosphatidylinositol-4-phosphate (PtdIns4P), whose ratio to phosphatidylinositol- 4,5-bisphosphate (PtdIns(4,5)P_2_) is usually 10 to 1. *In vivo* ^32^P_i_ incorporation studies have demonstrated a more rapid turnover of PtdIns(4,5)P_2_ levels, which together with its low abundance at the plasma membrane characterizes this compound as a signaling molecule (Munnik and Nielsen, 2011; Boss and Im, 2012). Indeed, this metabolite is produced by the activity of PtdIns4 5-kinases (PIP5K), whereas signal termination is achieved upon activation of 5′-phosphatidyilinositol phosphatases (5Ptase) (**Figure 2B**). The tight levels of PtdIns(4,5)P_2_ at the plasma membrane are also controlled by the activity of PHOSPHOLIPASE C (PLC), an enzyme that cleaves PtdIns(4,5)P_2_ into inositol-trisphosphate (InsP_3_) and 1,2-diacylglycerol (DAG). DAG can be further phosphorylated by diacylglycerol kinases (DGK), leading to the formation of phosphatidic acid (PA). Besides PI-specific PLC, plants also encode non-specific PLC (NPC) which can use phosphatidyl choline (PtdCho) or phosphatidyl ethanolamine (PtdE) to generate DAG and the corresponding phosphoalcohol (Wimalasekera et al., 2010). In summary, various kinases, phosphatases and lipases can metabolize several phosphoglycerolipids, and generate distinct pools of different phospholipid species. Thus, a tight regulation of phospholipid catabolism is crucial to determine adequate biological responses.

## Phosphoglycerolipids Distribution within a Cell

**FIGURE 3:**
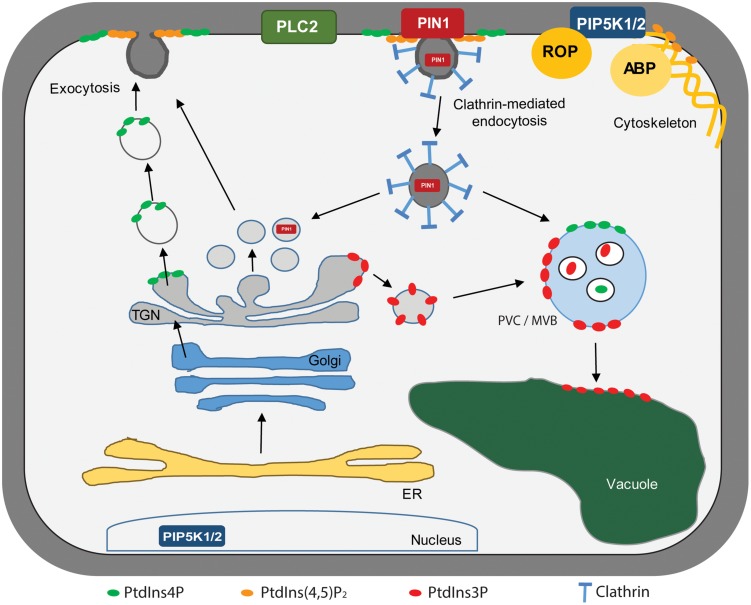
**Asymmetrical phosphoinositide distribution across the subcellular membranes.** Phosphoinositide-interacting proteins as well as phosphoinositide biosynthetic enzymes have been represented within a plant cell. PLC2, PHOSPHOLIPASE C 2; PIN1, PIN-FORMED 1; PIP5K1/2, PtdIns4P-5-KINASE 1/2; ROP, RHO OF PLANTS; ABP, ACTIN BINDING PROTEIN, TGN, Trans-Golgi Network; ER, Endoplasmic Reticulum; PVC/MVB, Prevacuolar Compartment/Multivesicular Body.

Cell compartmentalization by an endomembrane system was a great evolutionary advantage that allows the independent coexistence of multiple biochemical environments within one cell (Gabaldon and Pittis, 2015). Such subcellular compartments are enriched or depleted in specific phosphoglycerolipids, which constitute a hallmark for these organelles and contributes to define their identity. While phosphoglycerolipids’ biosynthesis mainly occurs in the endoplasmic reticulum (ER), live cell-imaging studies have shown an asymmetrical distribution of these lipid-derived metabolites across the diverse organelle membranes (**Figure 3**; Heilmann and Heilmann, 2015). To reach their final destination, phospholipids become incorporated into vesicles membranes within the ER to be further shuttled to other organelle’s membrane. Additionally, cytosolic PtdIns transfer proteins enable the rapid reallocation of these compounds between membranes, ensuring an unequal intracellular distribution across all subcellular compartments (Hurley and Meyer, 2001; Phillips et al., 2006). Furthermore, the enzymes involved in phospholipid inter-conversion display largely exclusive subcellular localization patterns, suggesting that the dynamic inter-organelle communication might be coordinated by the presence of specific phospholipids at the organelle’s membrane. Particularly, the subcellular distribution of phosphatidylinositol-3-phosphate (PtdIns3P), PtdIns4P, and PtdIns(4,5)P_2_ within plant cells have been described in the recent years. By using adapted PI-specific fluorescent biosensors containing well known PI-recognition domains developed from the animal research field, several studies have demonstrated the different distribution of these metabolites across the membranes (Balla, 2007). Analysis of PtdIns3P-specific FYVE-based reporter indicated that PtdIns3P is gradually distributed from late endosomes to the tonoplast, confirming a regulatory activity for this compound in protein trafficking toward the vacuole (Vermeer et al., 2006). On the contrary, PI-4-kinases are mainly localized in the *trans*-golgi network (TGN), even if their catalytic product PtdIns4P was additionally found at the plasma membrane (PM), implying the existence of a shuttle-mechanism toward this compartment (Tejos et al., 2014). Likewise, PtdIns(4,5)P_2_ decorated by the PLCδ1-PH-GFP reporter was predominantly observed at the PM of plant cells together with PLC2 and PI4P 5-kinases, indicating that PIs phosphorylated at the 4′ position are involved in regulating cellular trafficking from the Golgi to the PM (van Leeuwen et al., 2007; Ischebeck et al., 2008; Stenzel et al., 2008; Tejos et al., 2014; Kanehara et al., 2015). However, PtdIns(4,5)P_2_ has been also involved in endocytic pathways, in particular in the modulation of chlatrin-mediated endocytosis events (Ischebeck et al., 2013). Together, these findings suggest that PtdIns(4,5)P_2_ might act as a scaffold signal for PtdIns(4,5)P_2_ -interacting proteins, altering their intracellular localization and/or enzymatic activity (van Leeuwen et al., 2004; Heilmann, 2009). However, the prominent localization of *PIP5K1/2* in the nucleus suggests additional, yet-to-be described roles for PtdIns(4,5)P_2_ in the regulation of nuclear events, as it has been reported in the animal field (Drobak and Heras, 2002; Keune et al., 2011; Tejos et al., 2014). Beyond its role in controlling protein trafficking, PtdIns(4,5)P_2_ also modulates the association of the cytoskeleton to the PM (Braun et al., 1999; Dong et al., 2001). By interacting with diverse actin binding proteins (ABPs; **Figure 3**), this compound increases actin polymerization which in turn promotes a closer attachment of the cytoskeleton to the PM. Additionally, PtdIns(4,5)P_2_ has been reported to regulate ROP (Rho of plants) GTPases. When active, ROP variants are associated to the PM where they coordinate actin organization and membrane trafficking (Pleskot et al., 2012, 2014). Importantly, PIP5K physically interacts with ROP at the apical PM of pollen tubes, where counteracts the activity of Rho-GDI (Rho-guanine nucleotide dissociation inhibitor) (Ischebeck et al., 2011). Thus, PtdIns(4,5)P_2_ modulates actin dynamics by regulating the pool of membrane-localized ROP GTPases (Yalovsky et al., 2008; Ischebeck et al., 2011). Together, PI’s abilities to regulate diverse subcellular events advocate these lipid-derived molecules as ideal candidates to orchestrate the organelle redistribution observed during vascular cell differentiation.

## Phosphoglycerolipids and the Establishment of Procambial Tissue

**FIGURE 4:**
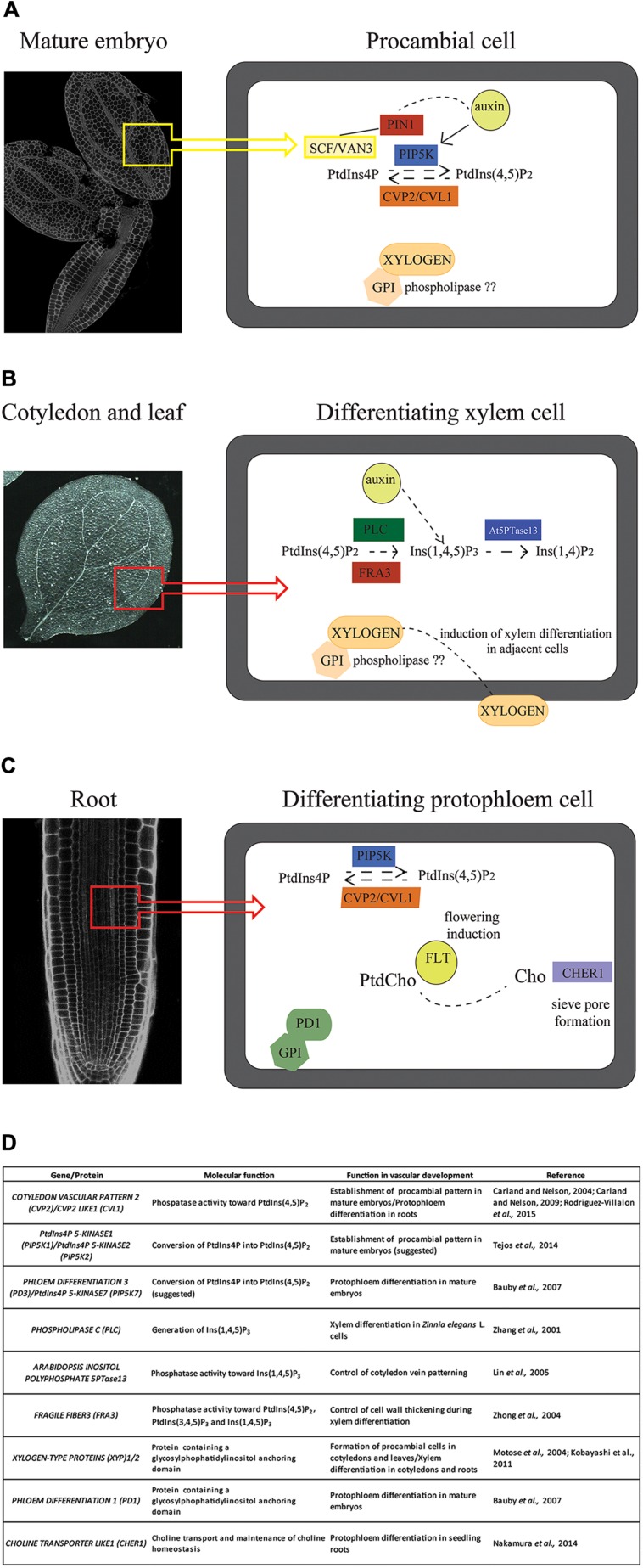
**Summarized function of phosphoglycerolipids in the regulation of vascular development.** **(A–C)** Putative molecular mechanisms regulating procambial cell fate acquisition in mature embryos **(A)**, xylem differentiation in leaf and cotyledon vascular cells **(B)** and protophloem differentiation in a root phloem cell **(C)**. **(D)** Table summarizing phosphoglycerolipd biosynthetic enzymes and phosphoglycerolipid-related proteins that modulate vascular development. PtdIns4P, phosphatidylinositol 4-phosphate; PtdInsP(4,5)P_2_, phosphatidylinositol 4,5-bisphosphate; Ins(1,4,5)P_3_, inositol 1,4,5 triphosphate; PtdCho, phosphatidylcholine; Cho, choline; SCF/VAN3, SCARFACE/VAN 3; PIN1, PIN-FORMED 1; PIP5K7, PtdIns4P-5-KINASE 7; CVP2, COTYLEDON VASCULAR PATTERN 2; CVL1, CVP2-LIKE 1; PLC2, PHOSPHOLIPASE C 2; GPI, Glycosylphosphatidylinositol anchor; FRA3, FRAGILE FIBER 3; At5PTase13, PHOSPHATIDYLINOSITOL 5′ PHOSPHATASE 13; FLT, FLOWERING LOCUS T; CHER1: CHOLINE TRANSPORTER LIKE 1; PD1, PHLOEM DIFFERENTIATION 1.

Our first notion about the regulatory role of phosphoglycerolipids in vascular development arose from the identification of *COTYLEDON VASCULAR PATTERN2 (CVP2)*, a 5PTase that exhibits phosphatase activity toward PtdIns(4,5)P_2_ (**Figure 2B**; Carland and Nelson, 2004, 2009). In *Arabidopsis*, the vascular system in cotyledons is composed of phloem and xylem tissues as well as by non-differentiated procambial cells (Sieburth and Deyholos, 2006; Scarpella and Helariutta, 2010; Schuetz et al., 2013). The position and cell fate acquisition of vascular precursor, the so-called procambial cells, are specified during embryogenesis, when the wide expression of the auxin exporter *PIN-FORMED1 (PIN1)* is restricted to certain population of ground cells toward which auxin will be canalized (Sachs, 1991; Mattsson et al., 1999; Sauer and Friml, 2004; Izhaki and Bowman, 2007). As germination proceeds, the differentiation of vascular bundles into phloem and xylem is completed, leading to the characteristic continuous and complex vein pattern observed in *Arabidopsis* cotyledons and leaves (Nelson and Dengler, 1997; Busse and Evert, 1999). The impact of PtdIns4P homeostasis on the control of this process was demonstrated by the phenotypical analysis of *cvp2* single mutants, whose discontinuous vein pattern in cotyledons and leaves are further compromised by knocking out *cvp2 like1 (cvl1)* activity (Carland and Nelson, 2009). Closer examination of *cvp2* mature embryos revealed that the disrupted vascular network observed in post-embryonic tissues is due to an impaired establishment of procambial patterning occurring at the embryonic stage (Carland et al., 1999; Carland and Nelson, 2004). Intriguingly, a similar phenotype was found in *pip5k1 pip5k2* leaves, where PIN1 polar localization in the developing vascular strands is lost and as result, most of the vascular strands fail to connect (Tejos et al., 2014). Furthermore, CVP2 and CVL1 activities are required to generate the specific PI-ligand of *SCARFACE/VASCULAR NETWORK DEFECTIVE3 (SCF/VAN3)* (**Figure 4A**), an ADP-ribosylation factor GTPase-activating protein (ARF GAP) which is localized at the TGN (Koizumi et al., 2005; Sieburth and Deyholos, 2006; Naramoto et al., 2009). Accordingly, *scarface* mutants exhibits an open vein phenotype identical to *cvp2 cvl1*, suggesting that they control procambial patterning by modulating intracellular vesicle transport and cell polarity in selected cells during leaf development (Carland and Nelson, 2009; Naramoto et al., 2009). Remarkably, *VAN3 ARF GAP* activity participates in the dynamic localization of PIN1 in root cortical cells, suggesting that PIs may be required to maintain auxin gradients during vascular development (**Figures 4A,D**; Berleth and Sachs, 2001; Sieburth and Deyholos, 2006; Scarpella et al., 2006).

## Phosphoglycerolipids and Xylem Differentiation

The importance of phospholipids in the progression of xylem development was highlighted by the discovery of xylogen, an extracellular proteoglycan containing a glycosylphosphatidylinositol (GPI)-anchoring domains (Motose et al., 2004; Kobayashi et al., 2011). The GPI anchor is made of one molecule of phosphatidylinositol to which a carbohydrate chain is linked through the 6′ hydroxyl of the inositol, and is linked to the protein through an ethanolamine phosphate moiety (Gruenberg, 2001). In addition to this domain, xylogen as well as the recently discovered XYLOGEN-TYPE PROTEINS (XYP) have non-specific lipid transfer protein sequences (Motose et al., 2004; Kobayashi et al., 2011). In particular, xylogen has been involved in pattern formation of procambial tissues as well as in the coordination of xylem differentiation (Motose et al., 2004). This notion is supported by the polar localization of this protein at the plasma membrane of differentiating cells, from where it is secreted to induce the differentiation of vascular uncommitted cells. As the GPI domain has been widely suggested to serve as an anchor of the proteins to the plasma membrane, it appears that this domain is responsible for the polar xylogen localization within the cell. However, xylogen was previously identified as a diffusible apoplastic factor in *Zinnia elegans* cells (Motose et al., 2001a,b), implying the existence of at least one phospholipase that cleaves the glycosylated protein and releases this factor to the apoplast, where it will promote the differentiation of adjacent cells (**Figure 4B**). Interestingly, genetic evidence has demonstrated the functional redundancy within the XYP family: while *xpy1* and *xpy2* single mutants exhibit continuous vein strands in the cotyledons, severe defects were found in *xpy1 xpy2* double mutants (Motose et al., 2004). Moreover, recent studies have shown a specific vascular localization of other members of this family such as *XPY7;* however, further research will be necessary to elucidate the potential role of this gene during vascular tissue formation (Kobayashi et al., 2011).

Nevertheless, other PIs such Ins(1,4,5)P_3_ has been involved in the regulation of xylem differentiation. In particular, an increase in PLC activity as well as in the concentration of its catalytic product Ins(1,4,5)P_3_ are required during the early stages of xylem differentiation, as indicated by the reduction of differentiated xylem cells upon application of PLC inhibitors (Zhang et al., 2002). Furthermore, auxin is required to induce Ins(1,4,5)P_3_ production, as demonstrated by the restoration of vascular defects in *Arabidopsis inositol polyphosphate 5PTase13*-1 upon exogenous auxin application (Zhang et al., 2002; Lin et al., 2005). Since this hormone induces the transcriptional activation of *PIP5K1/2*, it appears that auxin increases PI metabolic flux to control its own directional transport (Zhang et al., 2002; Tejos et al., 2014). However, whether PIs are involved in regulating organelle disintegration, cytoskeleton reorganization or protein trafficking required to sustain xylem differentiation has not been yet described. Noteworthy, studies in *fragile fiber 3*, a 5PTase with high affinity toward PtdIns(4,5)P_2_, revealed that the latter is required for actin organization and cell wall formation in stem fiber cells (**Figures 4B,D**; Zhong et al., 2004). Therefore, it is tempting to speculate that PtdIns(4,5)P_2_ can regulate xylem differentiation by modulating cytoskeleton rearrangement.

## Phosphoglycerolipids and Phloem Differentiation

Unlike xylem differentiation, very little is known about the underlying molecular mechanisms regulating protophloem differentiation programs. Our current understanding about phosphoglycerolipids involvement in the control of this process emerged by several studies performed in *Arabidopsis* roots. The initial identification of two gene-trap mutants named *PHLOEM DIFFERENTIATION 1-3* (PD1 and PD3) established a putative regulatory role for this compounds in the regulation of protophloem differentiation (Bauby et al., 2007). While PD1 encodes a putative GPI-anchored protein, PD3 was predicted to code for the PtdIns(4,5)P_2_-biosynthetic enzyme *PIP5K7* (**Figures 4C,D**). Consistent with a PI role in protophloem differentiation, a recent study has shown that *CVP2* and *CVL1* are specifically expressed in root phloem cells (Rodriguez-Villalon et al., 2015). Indeed, a tight balance of CVP2-controlled PtdIns(4,5)P_2_ levels has a strong impact on the differentiation program of this tissue, as revealed by the presence of undifferentiated cells in *cvp2 cvl1* protophloem strands. Paradoxically, a similar phenotype was described while constitutively expressing *CVP2*, reinforcing the notion that a tight PtdIns4P / PtdIns(4,5)P_2_ balance is crucial to ensure an optimal protophloem differentiation (**Figures 4C,D**; Rodriguez-Villalon et al., 2015). Furthermore, PIP5K1 as well as its catalytic product PtdIns(4,5)P_2_ have been shown to be distributed polarly in procambium root cells, suggesting that these metabolites might be involved in regulation of asymmetric vesicle trafficking occurring during vascular development (Tejos et al., 2014). Moreover, a skewed PtdIns4P/PtdIns(4,5)P_2_ ratio has a strong impact on phloem conductivity, similar to the reduced phloem functionality described when choline homeostasis is compromised (Dettmer et al., 2014; Rodriguez-Villalon et al., 2015). In particular, deficient activity of the recently described *CHOLINE TRANSPORTER LIKE1 (CHER1)* affects tissue conductivity by decreasing the number of sieve pores present in the cells walls of adjacent phloem cells (**Figures 4C,D**; Dettmer et al., 2014). Interestingly, a choline-derived metabolite, PtdCho has recently been demonstrated to regulate the activity of the phloem-mobile *FLT* factor. In order to induce flowering, FLT is transported to the nucleus of the shoot meristematic cells where it binds diurnal PtdCho species, integrating environmental signal to the internal clock of the plant (Nakamura et al., 2014). In particular, daily environmental changes in light or temperature have shown to modify PtdCho species accumulated at the PM. Thus, by specifically binding diurnal PtCho-derivatives, plants have evolved a sophisticated mechanism to regulate circadian physiological responses such as flowering time.

## Concluding Remarks and Future Perspectives

Far from our classical view of phosphoglycerolipids as structural membrane components, these metabolites have begun to emerge as crucial signaling compounds in plant development. While the repertoire of cellular processes known to be directly or indirectly controlled by this class of lipids has now dramatically expanded in the animal field, knowledge about their specific functions in plants is just at its infancy. However, as it is clear from this overview, the presence of several PI and choline-derivatives in vascular exudates correlates with a clear regulatory role of these lipids in vascular formation and functionality. Their rapid turnover as well as their abilities in regulating cytoskeleton organization and protein trafficking advocate these metabolites as ideal candidates to regulate complex morphogenetic processes such as plant vascular differentiation. However, present studies did not overpass simple morphological tissue description of phospholipid biosynthetic mutants. Therefore, future studies are required to elucidate the specific regulatory role of each metabolite in the control of xylem and phloem differentiation. Nevertheless, different lines of evidence have demonstrated that PIs are involved in the establishment and maintenance of auxin gradients, essential to determine vascular patterning and differentiation. By regulating chlatrin-mediated endocytosis events, PtdIns(4,5)P_2_ modulates the polar distribution of the auxin efflux carrier PIN1 (Ischebeck et al., 2013; Tejos et al., 2014). Therefore, a central question is whether PtdIns(4,5)P_2_ mediates endocytic or secretory pathways of other polarly distributed vascular proteins, or if this mechanism is exclusive for PIN proteins. Additionally, the defective protophloem differentiation observed in *cvp2 cvl1* mutants has been associated with a reduced post-embryonic root growth and higher lateral root density. The presence of some undifferentiated cells in root protophloem strands prevents the shoot-to-root delivery of auxin, which is accumulated at the maturation zone of the root promoting an enhanced lateral root emergence (Rodriguez-Villalon et al., 2015). Similarly, other mutants with perturbed PtdIns4P / PtdIns(4,5)P_2_ homeostasis such as *pip5k1 pip5k2, plc2*, and *pi4kIIIβ1β2* mutants exhibit identical root phenotypes (Ischebeck et al., 2013; Novakova et al., 2014; Sasek et al., 2014; Tejos et al., 2014), reinforcing the notion that a tight PtdIns4P and PtdIns(4,5)P_2_ balance is crucial to sustain optimal root growth. Interestingly, inactivation of salicylic acid (SA) signaling pathway restores a normal shoot growth but not normal root growth in *pi4kIIIβ1β2*, indicating that the higher SA accumulation observed in these plants is not responsible for the reduced post-embryonic root growth (Sasek et al., 2014). Thus, further experiments are required to unravel whether the short root phenotype exhibited by these mutants is the result of a defective protophloem differentiation or whether it exist a specific PI-dependent mechanism yet- to-be described.

Overall, plant PIs have emerged as pivotal players in controlling plant vascular development. The future elucidation of PI-dependent signaling networks and their crosstalk with several signaling pathways will broaden our knowledge of such fascinating processes. Furthermore, a future challenge will be to decipher how PtdCho and its derivatives link the internal plant circadian clock with environmental-mediated responses. In summary, while is nowadays clear that phosphoglycerolipids are crucial for many aspects of vascular formation, the underlying regulatory mechanisms remain still poorly understood.

## Author Contributions

BG and AR-V wrote the manuscript.

## Conflict of Interest Statement

The authors declare that the research was conducted in the absence of any commercial or financial relationships that could be construed as a potential conflict of interest. The reviewer CG and handling Editor declared their shared affiliation, and the handling Editor states that the process nevertheless met the standards of a fair and objective review.

## References

[B1] Balla T. (2007). Imaging and manipulating phosphoinositides in living cells. *J. Physiol. Lond.* 582 927–937. 10.1113/jphysiol.2007.132795 17395624 PMC2075240

[B2] Bauby H. Divol F. Truernit E. Grandjean O. Palauqui J. C. (2007). Protophloem differentiation in early *Arabidopsis thaliana* development. *Plant Cell Physiol.* 48 97–109. 10.1093/pcp/pcl045 17135286

[B3] Berleth T. Sachs T. (2001). Plant morphogenesis: long-distance coordination and local patterning. *Curr. Opin. Plant Biol.* 4 57–62. 10.1016/S1369-5266(00)00136-9 11163169

[B4] Boss W. F. Im Y. J. (2012). Phosphoinositide signaling. *Annu. Rev. Plant Biol.* 63 409–429. 10.1146/annurev-arplant-042110-103840 22404474

[B5] Braun M. Baluska F. Von Witsch M. Menzel D. (1999). Redistribution of actin, profilin and phosphatidylinositol-4,5-bisphosphate in growing and maturing root hairs. *Planta* 209 435–443. 10.1007/s004250050746 10550624

[B6] Busse J. S. Evert R. F. (1999). Vascular differentiation and transition in the seedling of *Arabidopsis thaliana* (Brassicaceae). *Int. J. Plant Sci.* 160 241–251. 10.1086/314117

[B7] Carland F. M. Berg B. L. Fitzgerald J. N. Jinamornphongs S. Nelson T. Keith B. (1999). Genetic regulation of vascular tissue patterning in *Arabidopsis.* *Plant Cell* 11 2123–2137. 10.1105/tpc.11.11.2123 10559439 PMC144128

[B8] Carland F. M. Nelson T. (2004). Cotyledon vascular pattern2-mediated inositol (1,4,5) triphosphate signal transduction is essential for closed venation patterns of *Arabidopsis* foliar organs. *Plant Cell* 16 1263–1275. 10.1105/tpc.021030 15100402 PMC423214

[B9] Carland F. M. Nelson T. (2009). CVP2- and CVL1-mediated phosphoinositide signaling as a regulator of the ARF GAP SFC/VAN3 in establishment of foliar vein patterns. *Plant J.* 59 895–907. 10.1111/j.1365-313X.2009.03920.x 19473324

[B10] Dettmer J. Ursache R. Campilho A. Miyashima S. Belevich I. O’regan S. (2014). CHOLINE TRANSPORTER-LIKE1 is required for sieve plate development to mediate long-distance cell-to-cell communication. *Nat. Commun.* 5:4276 10.1038/ncomms5276 25008948

[B11] Di Paolo G. De Camilli P. (2006). Phosphoinositides in cell regulation and membrane dynamics. *Nature* 443 651–657. 10.1038/nature05185 17035995

[B12] Dong C. H. Xia G. X. Hong Y. Ramachandran S. Kost B. Chua N. H. (2001). ADF proteins are involved in the control of flowering and regulate F-actin organization, cell expansion, and organ growth in *Arabidopsis*. *Plant Cell* 13 1333–1346. 10.1105/tpc.13.6.1333 11402164 PMC135580

[B13] Drobak B. K. Heras B. (2002). Nuclear phosphoinositides could bring FYVE alive. *Trends Plant Sci.* 7 132–138. 10.1016/S1360-1385(01)02213-0 11906837

[B14] Furuta K. M. Yadav S. R. Lehesranta S. Belevich I. Miyashima S. Heo J. O. (2014). Plant development. *Arabidopsis* NAC45/86 direct sieve element morphogenesis culminating in enucleation. *Science* 345 933–937. 10.1126/science.1253736 25081480

[B15] Gabaldon T. Pittis A. A. (2015). Origin and evolution of metabolic sub-cellular compartmentalization in eukaryotes. *Biochimie* 119 262–268. 10.1016/j.biochi.2015.03.021 25869000 PMC4678951

[B16] Gruenberg J. (2001). The endocytic pathway: a mosaic of domains. *Nat. Rev. Mol. Cell Biol.* 2 721–730. 10.1038/35096054 11584299

[B17] Guelette B. S. Benning U. F. Hoffmann-Benning S. (2012). Identification of lipids and lipid-binding proteins in phloem exudates from *Arabidopsis thaliana*. *J. Exp. Bot.* 63 3603–3616. 10.1093/jxb/ers028 22442409 PMC3388829

[B18] Heilmann I. (2009). Using genetic tools to understand plant phosphoinositide signalling. *Trends Plant Sci.* 14 171–179. 10.1016/j.tplants.2008.12.002 19217341

[B19] Heilmann M. Heilmann I. (2015). Plant phosphoinositides-complex networks controlling growth and adaptation. *Biochim. Biophys. Acta* 1851 759–769. 10.1016/j.bbalip.2014.09.018 25280638

[B20] Hurley J. H. Meyer T. (2001). Subcellular targeting by membrane lipids. *Curr. Opin. Cell Biol.* 13 146–152. 10.1016/S0955-0674(00)00191-5 11248547

[B21] Ischebeck T. Stenzel I. Heilmann I. (2008). Type B phosphatidylinositol-4-phosphate 5-kinases mediate *Arabidopsis* and *Nicotiana tabacum* pollen tube growth by regulating apical pectin secretion. *Plant Cell* 20 3312–3330. 10.1105/tpc.108.059568 19060112 PMC2630452

[B22] Ischebeck T. Stenzel I. Hempel F. Jin X. Mosblech A. Heilmann I. (2011). Phosphatidylinositol-4,5-bisphosphate influences Nt-Rac5-mediated cell expansion in pollen tubes of *Nicotiana tabacum*. *Plant J.* 65 453–468. 10.1111/j.1365-313X.2010.04435.x 21265898

[B23] Ischebeck T. Werner S. Krishnamoorthy P. Lerche J. Meijon M. Stenzel I. (2013). Phosphatidylinositol 4,5-bisphosphate influences PIN polarization by controlling clathrin-mediated membrane trafficking in *Arabidopsis*. *Plant Cell* 25 4894–4911. 10.1105/tpc.113.116582 24326589 PMC3903994

[B24] Izhaki A. Bowman J. L. (2007). KANADI and class III HD-Zip gene families regulate embryo patterning and modulate auxin flow during embryogenesis in *Arabidopsis*. *Plant Cell* 19 495–508. 10.1105/tpc.106.047472 17307928 PMC1867338

[B25] Janda M. Planchais S. Djafi N. Martinec J. Burketova L. Valentova O. (2013). Phosphoglycerolipids are master players in plant hormone signal transduction. *Plant Cell Rep.* 32 839–851. 10.1007/s00299-013-1399-0 23471417

[B26] Kanehara K. Yu C. Y. Cho Y. Cheong W. F. Torta F. Shui G. (2015). Arabidopsis AtPLC2 Is a Primary phosphoinositide-specific phospholipase c in phosphoinositide metabolism and the endoplasmic reticulum stress response. *PLoS Genet.* 11:e1005511 10.1371/journal.pgen.1005511 PMC4581737 26401841

[B27] Keune W. Bultsma Y. Sommer L. Jones D. Divecha N. (2011). Phosphoinositide signalling in the nucleus. *Adv. Enzyme Regul.* 51 91–99. 10.1016/j.advenzreg.2010.09.009 21035491

[B28] Kobayashi Y. Motose H. Iwamoto K. Fukuda H. (2011). Expression and genome-wide analysis of the xylogen-type gene family. *Plant Cell Physiol.* 52 1095–1106. 10.1093/pcp/pcr060 21558309

[B29] Koizumi K. Naramoto S. Sawa S. Yahara N. Ueda T. Nakano A. (2005). VAN3 ARF–GAP-mediated vesicle transport is involved in leaf vascular network formation. *Development* 132 1699–1711. 10.1242/dev.01716 15743878

[B30] Lin W. H. Wang Y. Mueller-Roeber B. Brearley C. A. Xu Z. H. Xue H. W. (2005). At5PTase13 modulates cotyledon vein development through regulating auxin homeostasis. *Plant Physiol.* 139 1677–1691. 10.1104/pp.105.067140 16299182 PMC1310551

[B31] Lucas W. J. Groover A. Lichtenberger R. Furuta K. Yadav S. R. Helariutta Y. (2013). The plant vascular system: evolution, development and functions. *J. Integr. Plant Biol.* 55 294–388. 10.1111/jipb.12041 23462277

[B32] Mattsson J. Sung Z. R. Berleth T. (1999). Responses of plant vascular systems to auxin transport inhibition. *Development* 126 2979–2991. 10357941 10.1242/dev.126.13.2979

[B33] Motose H. Fukuda H. Sugiyama M. (2001a). Involvement of local intercellular communication in the differentiation of zinnia mesophyll cells into tracheary elements. *Planta* 213 121–131. 10.1007/s004250000482 11523648

[B34] Motose H. Sugiyama M. Fukuda H. (2001b). An arabinogalactan protein(s) is a key component of a fraction that mediates local intercellular communication involved in tracheary element differentiation of zinnia mesophyll cells. *Plant Cell Physiol.* 42 129–137. 10.1093/pcp/pce014 11230566

[B35] Motose H. Sugiyama M. Fukuda H. (2004). A proteoglycan mediates inductive interaction during plant vascular development. *Nature* 429 873–878. 10.1038/nature02613 15215864

[B36] Munnik T. Nielsen E. (2011). Green light for polyphosphoinositide signals in plants. *Curr. Opin. Plant Biol.* 14 489–497. 10.1016/j.pbi.2011.06.007 21775194

[B37] Nakamura Y. Andres F. Kanehara K. Liu Y. C. Dormann P. Coupland G. (2014). *Arabidopsis* florigen FT binds to diurnally oscillating phospholipids that accelerate flowering. *Nat. Commun.* 5:3553 10.1038/ncomms4553 PMC3988816 24698997

[B38] Naramoto S. Sawa S. Koizumi K. Uemura T. Ueda T. Friml J. (2009). Phosphoinositide-dependent regulation of VAN3 ARF-GAP localization and activity essential for vascular tissue continuity in plants. *Development* 136 1529–1538. 10.1242/dev.030098 19363154

[B39] Nelson T. Dengler N. (1997). Leaf vascular pattern formation. *Plant Cell* 9 1121–1135. 10.1105/tpc.9.7.1121 12237378 PMC156985

[B40] Novakova P. Hirsch S. Feraru E. Tejos R. Van Wijk R. Viaene T. (2014). SAC phosphoinositide phosphatases at the tonoplast mediate vacuolar function in *Arabidopsis.* *Proc. Natl. Acad. Sci. U.S.A.* 111 2818–2823. 10.1073/pnas.1324264111 24550313 PMC3932866

[B41] Oparka K. J. Turgeon R. (1999). Sieve elements and companion cells-traffic control centers of the phloem. *Plant Cell* 11 739–750. 10.2307/3870896 10213790 PMC144213

[B42] Phillips S. E. Vincent P. Rizzieri K. E. Schaaf G. Bankaitis V. A. Gaucher E. A. (2006). The diverse biological functions of phosphatidylinositol transfer proteins in eukaryotes. *Crit. Rev. Biochem. Mol. Biol.* 41 21–49. 10.1080/10409230500519573 16455519

[B43] Pleskot R. Pejchar P. Staiger C. J. Potocky M. (2014). When fat is not bad: the regulation of actin dynamics by phospholipid signaling molecules. *Front. Plant Sci.* 5:5 10.3389/fpls.2014.00005 PMC3899574 24478785

[B44] Pleskot R. Pejchar P. Zarsky V. Staiger C. J. Potocky M. (2012). Structural Insights into the inhibition of actin-capping protein by interactions with phosphatidic acid and phosphatidylinositol (4,5)-Bisphosphate. *PLoS Comput. Biol.* 8:e1002765 10.1371/journal.pcbi.1002765 PMC3486809 23133367

[B45] Rodriguez-Villalon A. Gujas B. Kang Y. H. Breda A. S. Cattaneo P. Depuydt S. (2014). Molecular genetic framework for protophloem formation. *Proc. Natl. Acad. Sci. U.S.A.* 111 11551–11556. 10.1073/pnas.1407337111 25049386 PMC4128119

[B46] Rodriguez-Villalon A. Gujas B. Van Wijk R. Munnik T. Hardtke C. S. (2015). Primary root protophloem differentiation requires balanced phosphatidylinositol-4,5-biphosphate levels and systemically affects root branching. *Development* 142 1437–1446. 10.1242/dev.118364 25813544

[B47] Ruiz-Medrano R. Xoconostle-Cazares B. Lucas W. J. (2001). The phloem as a conduit for inter-organ communication. *Curr. Opin. Plant Biol.* 4 202–209. 10.1016/S1369-5266(00)00162-X 11312130

[B48] Sachs T. (1991). Cell polarity and tissue patterning in plants. *Development* 2 83–93.

[B49] Sasek V. Janda M. Delage E. Puyaubert J. Guivarc’h A. Maseda E. L. (2014). Constitutive salicylic acid accumulation in pi4kIII beta 1 beta 2 *Arabidopsis* plants stunts rosette but not root growth. *New Phytol.* 203 805–816. 10.1111/nph.12822 24758581

[B50] Sauer M. Friml J. (2004). In vitro culture of *Arabidopsis* embryos within their ovules. *Plant J.* 40 835–843. 10.1111/j.1365-313X.2004.02248.x 15546365

[B51] Scarpella E. Helariutta Y. (2010). Vascular pattern formation in plants. *Curr. Top. Dev. Biol.* 91 221–265. 10.1016/S0070-2153(10)91008-9 20705184

[B52] Scarpella E. Marcos D. Friml J. Berleth T. (2006). Control of leaf vascular patterning by polar auxin transport. *Genes Dev.* 20 1015–1027. 10.1101/gad.1402406 16618807 PMC1472298

[B53] Schuetz M. Smith R. Ellis B. (2013). Xylem tissue specification, patterning, and differentiation mechanisms. *J. Exp. Bot.* 64 11–31. 10.1093/jxb/ers287 23162114

[B54] Sieburth L. E. Deyholos M. K. (2006). Vascular development: the long and winding road. *Curr. Opin. Plant Biol.* 9 48–54. 10.1016/j.pbi.2005.11.008 16332447

[B55] Stadler R. Wright K. M. Lauterbach C. Amon G. Gahrtz M. Feuerstein A. (2005). Expression of GFP-fusions in *Arabidopsis* companion cells reveals non-specific protein trafficking into sieve elements and identifies a novel post-phloem domain in roots. *Plant J.* 41 319–331. 10.1111/j.1365-313X.2004.02298.x 15634207

[B56] Stenzel I. Ischebeck T. Konig S. Holubowska A. Sporysz M. Hause B. (2008). The type B phosphatidylinositol-4-phosphate 5-kinase 3 is essential for root hair formation in *Arabidopsis thaliana*. *Plant Cell* 20 124–141. 10.1105/tpc.107.052852 18178770 PMC2254927

[B57] Tejos R. Sauer M. Vanneste S. Palacios-Gomez M. Li H. Heilmann M. (2014). Bipolar plasma membrane distribution of phosphoinositides and their requirement for auxin-mediated cell polarity and patterning in *Arabidopsis*. *Plant Cell* 26 2114–2128. 10.1105/tpc.114.126185 24876254 PMC4079372

[B58] van Leeuwen W. Okresz L. Bogre L. Munnik T. (2004). Learning the lipid language of plant signalling. *Trends Plant Sci.* 9 378–384. 10.1016/j.tplants.2004.06.008 15358268

[B59] van Leeuwen W. Vermeer J. E. Gadella T. W. Jr. Munnik T. (2007). Visualization of phosphatidylinositol 4,5-bisphosphate in the plasma membrane of suspension-cultured tobacco BY-2 cells and whole *Arabidopsis* seedlings. *Plant J.* 52 1014–1026. 10.1111/j.1365-313X.2007.03292.x 17908156

[B60] Vermeer J. E. Van Leeuwen W. Tobena-Santamaria R. Laxalt A. M. Jones D. R. Divecha N. (2006). Visualization of PtdIns3P dynamics in living plant cells. *Plant J.* 47 687–700. 10.1111/j.1365-313X.2006.02830.x 16856980

[B61] Wimalasekera R. Pejchar P. Holk A. Martinec J. Scherer G. F. (2010). Plant phosphatidylcholine-hydrolyzing phospholipases C NPC3 and NPC4 with roles in root development and brassinolide signaling in *Arabidopsis thaliana*. *Mol. Plant* 3 610–625. 10.1093/mp/ssq005 20507939

[B62] Yalovsky S. Bloch D. Sorek N. Kost B. (2008). Regulation of membrane trafficking, cytoskeleton dynamics, and cell polarity by ROP/RAC GTPases. *Plant Physiol.* 147 1527–1543. 10.1104/pp.108.122150 18678744 PMC2492628

[B63] Yoo S. C. Chen C. Rojas M. Daimon Y. Ham B. K. Araki T. (2013). Phloem long-distance delivery of FLOWERING LOCUS T (FT) to the apex. *Plant J.* 75 456–468. 10.1111/tpj.12213 23607279

[B64] Zhang X. G. Cote G. G. Crain R. C. (2002). Involvement of phosphoinositide turnover in tracheary element differentiation in *Zinnia elegans* L. cells. *Planta* 215 312–318. 10.1007/s00425-002-0739-z 12029481

[B65] Zhong R. Burk D. H. Morrison W. H. III Ye Z. H. (2004). FRAGILE FIBER3, an *Arabidopsis* gene encoding a type II inositol polyphosphate 5-phosphatase, is required for secondary wall synthesis and actin organization in fiber cells. *Plant Cell* 16 3242–3259. 10.1105/tpc.104.027466 15539468 PMC535871

